# Identification and classification of innexin gene transcripts in the central nervous system of the terrestrial slug *Limax valentianus*

**DOI:** 10.1371/journal.pone.0244902

**Published:** 2021-04-15

**Authors:** Hisayo Sadamoto, Hironobu Takahashi, Suguru Kobayashi, Hirooki Kondoh, Hiroshi Tokumaru

**Affiliations:** 1 Faculty of Pharmaceutical Sciences at Kagawa Campus, Tokushima Bunri University, Shido, Sanuki-City, Kagawa, Japan; 2 Faculty of Pharmaceutical Sciences, Tokushima Bunri University, Yamashiro-cho, Tokushima, Japan; Tokai University, JAPAN

## Abstract

Intercellular gap junction channels and single-membrane channels have been reported to regulate electrical synapse and the brain function. Innexin is known as a gap junction-related protein in invertebrates and is involved in the formation of intercellular gap junction channels and single-cell membrane channels. Multiple isoforms of innexin protein in each species enable the precise regulation of channel function. In molluscan species, sequence information of innexins is still limited and the sequences of multiple innexin isoforms have not been classified. This study examined the innexin transcripts expressed in the central nervous system of the terrestrial slug *Limax valentianus* and identified 16 transcripts of 12 innexin isoforms, including the splicing variants. We performed phylogenetic analysis and classified the isoforms with other molluscan innexin sequences. Next, the phosphorylation, N-glycosylation, and S-nitrosylation sites were predicted to characterize the innexin isoforms. Further, we identified 16 circular RNA sequences of nine innexin isoforms in the central nervous system of *Limax*. The identification and classification of molluscan innexin isoforms provided novel insights for understanding the regulatory mechanism of innexin in this phylum.

## Introduction

Gap junction channels are formed by docking of single-cell membrane channels between adjacent cells, and that allow intercellular communication via the exchange of small molecules such as ions, nucleotides, small peptides, and micro RNA (miRNA). Previous studies have indicated that the gap junction-related proteins are involved in the regulation of brain function. Neuronal gap junctions work as electrical synapses and exhibit plasticity [1, 2]. In addition, single-cell membrane channels, which do not form intercellular channels, are also involved in neuroplasticity through their interaction with chemical synapses [2, 3]. The channel properties of gap junction-related proteins are regulated by protein modifications such as N-glycosylation, S-nitrosylation, and phosphorylation [4–7].

As gap junction-related proteins in animal species, three protein families, innexin in invertebrates, connexin and pannexin in vertebrates, are known. These proteins share structural features that are characterized by four transmembrane domains, two extracellular loops, and intracellular N- and C-terminal domains [8]. The hexamers, heptamers, or octamers of these proteins form a single-cell membrane channel [9, 10]. Additionally, multiple isoforms of these proteins contribute to the specific regulation of channel properties and intercellular docking of single-cell membrane channels. Previous studies have also reported the differential characteristics of the three protein families [11, 12]. Vertebrate connexin proteins form both single-cell membrane channels and intercellular gap junctions. On the other hand, vertebrate pannexin proteins form only single-cell membrane channels *in vivo* as glycosylation at their extracellular domain inhibits the formation of intercellular gap junction channels. In invertebrates, innexin is the only protein family that exhibits the functions of both connexin and pannexin.

To start the elucidation of the regulatory function of gap junction-proteins in the brains, we here focused on innexin in molluscan species. In invertebrate neurophysiological research, molluscs are useful animal species because of their large, identifiable neurons. Neural circuit studies directly related to behavioral changes have been conducted, and electrical synapses between specific neurons have been studied [13–15]. For instance, electrical synapses have been demonstrated to exist and play important roles in feeding and withdrawal behaviors in the gastropod mollusc *Lymnaea stagnalis* [16–19]. The gastropod mollusc, terrestrial slug *Limax valentianus* is used as a model animal for studying the neural mechanism of learning and memory [20]. Especially, the olfactory memory of *Limax* has been well investigated [21, 22] and electrical synapses are involved in the neural mechanism of olfactory detection [23, 24].

Despite the many research on neural circuit identification and electrical synapses, there are limited studies on the molluscan innexin genes. Early studies identified two putative gap junction protein genes in the pteropod mollusc *Clione limacina* [25], and demonstrated that the injection of mRNA of one gene altered the electrical coupling between the identified neurons [26]. Recently, eight innexin gene transcripts were identified in *Lymnaea stagnalis* using transcriptome data [27]. Moreover, several studies have performed genomic and transcriptome analyses of various molluscan species [28–32], however, identified innexin homologs, including multiple isoforms, have not been classified.

This study aimed to identify innexin gene transcripts in the central nervous system (CNS) using the terrestrial slug *Limax valentianus*. We comparative and phylogenetic analyses of the predicted amino acid sequences of molluscan species were performed to classify the innexin homologs. We here examined the characterization of molluscan innexin isoforms, and first reported circular RNAs (circRNAs) expression of gap junction-related protein in the CNS. Our findings will facilitate future research on the regulatory mechanisms of gap junction-related proteins in the CNS of molluscan species.

## Methods

### Animals

All experiments were performed using the terrestrial slugs *Limax valentianus* at 3–4 months post-hatching. The animals were maintained under laboratory conditions at 19°C with a 12-h light/dark cycle. They were fed with humidified powder mixture containing the following: 520 g rat chow (Oriental Yeast, Tokyo, Japan), 500 g potato starch (Hokuren, Hokkaido, Japan), and 21 g vitamin mixture (AIN-76, Oriental Yeast). For the CNS isolation, slugs were anesthetized by a body cavity injection of Mg^2+^ buffer solution containing the following (in mM): 60.0 MgCl_2_, 5.0 glucose, and 5.0 HEPES, pH 7.0. The CNS were isolated in ice-cold high-Mg^2+^ saline containing the following (in mM): 35.0 NaCl, 2.0 KCl, 28.0 MgCl_2_, 4.9 CaCl_2_, 5.0 glucose, and 5.0 HEPES, pH 7.0. The isolated ganglia were transferred to a dish filled with a *Limax* saline solution containing the following (in mM): 70.0 NaCl, 2.0 KCl, 4.9 CaCl_2_, 4.7 MgCl_2_, 5.0 glucose, and 5.0 HEPES, pH 7.0. The isolated CNS was frozen in liquid nitrogen for RNA extraction.

### Polymerase Chain Reaction (PCR)

RNA was extracted from the dissected CNS of three animals using the Nucleospin RNA XS kit and treated with DNAse I (Macherey & Nagel, Düren, Germany). Total RNA (100 ng) was reverse-transcribed using M-MLV reverse transcriptase (Invitrogen, Carlsbad, CA) and random primers, following the manufacturer’s instructions. The cDNA was subjected to PCR using ExTaq DNA polymerase (Takara Co., Otsu, Japan) or PrimeSTAR GXL DNA polymerase kit (Takara Co.). The sequence-specific primers for *Limax* innexin mRNAs were designed based on the innexin homolog sequences identified through transcriptome shotgun assembly of *Limax valentianus* (paper in preparation). For amplifying circRNA, divergent primers were designed based on the verified complementary DNA (cDNA) sequences. The primer sequences are listed in supporting information (S1 Table). The amplicons were subcloned into the TOPO™ vector (Invitrogen) and subjected to nucleotide sequencing analysis.

### Protein sequence analyses of predicted innexin homologs

Phylogenetic analysis was performed using the available molluscan innexin sequences in the Swissprot, Genbank, and Refseq databases with basic local alignment search tool (BLAST). Multiple sequence alignment of molluscan innexin homologs was performed using MUSCLE. The phylogenetic tree was constructed using the maximum likelihood method with MEGAX software [33].

The potential phosphorylation sites of protein kinase C (PKC), protein kinase A (PKA), protein kinase G (PKG), casein kinase 1 (CK1), casein kinase 2 (CK2), proto-oncogene tyrosine-protein kinase Src (SRC), P34cdc (cdc2), calmodulin-dependent protein kinase (CaMK), and mitogen-activated protein kinase (p38MAPK) were predicted using NetPhos 3.1 Server [34]. Potential S-nitrosylation and N-glycosylation sites were predicted using GPS-SNO1.0 [35] and NetNGlyc 1.0 Server analysis (http://sno.biocuckoo.org/ and http://www.cbs.dtu.dk/services/NetNGlyc/), respectively. The consensus peptide sequence for N-glycosylation (Asn-X-Ser/Thr; X is not Pro) is known to be conserved in both vertebrates and invertebrates [36, 37].

## Results

### Identification of *Limax* innexin homologs

To identify the gene transcripts of gap junction proteins in the *Limax* CNS, a local BLASTX search was performed for our transcriptome data (paper in preparation) using sequences of *Aplysia* innexin homologs. The cDNA sequences were cloned and subjected to nucleotide sequencing. In this study, we identified 16 *Limax* innexin homologs with the entire coding domain sequence (CDS) (*Limax* innexin 1–11 including spliced isoforms) and partial CDS of *Limax* innexin 12 (accession numbers LC595664–LC595679).

**Fig 1:**
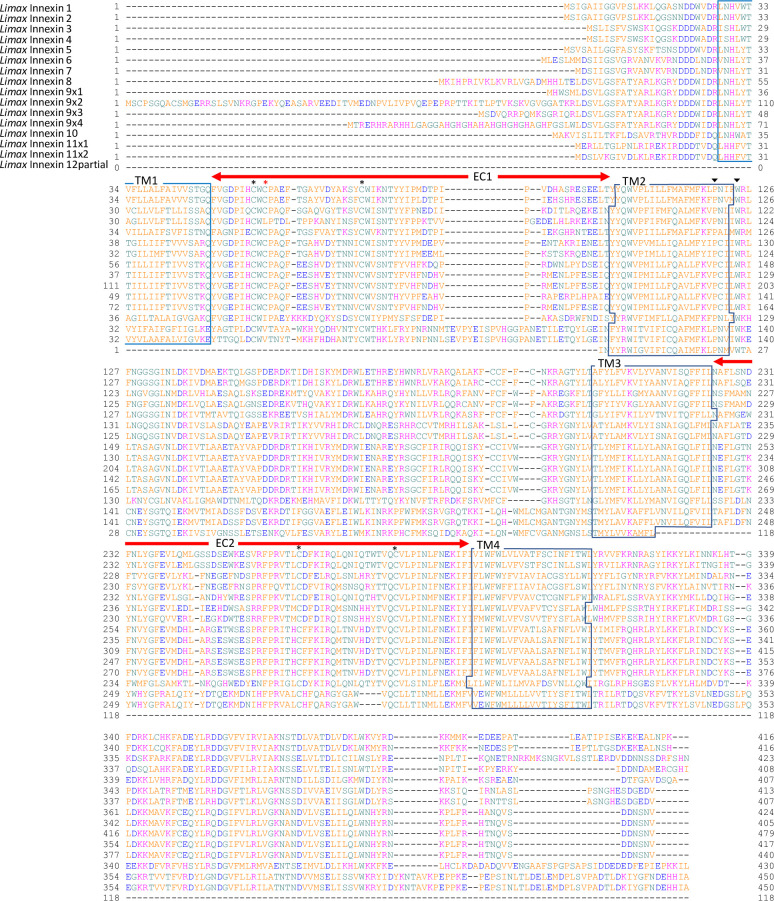
*Limax* innexin transcripts expressed in the central nervous system. Alignment of deduced amino acid sequences of the *Limax* innexin homologs identified in the transcriptome of the central nervous system. *Limax* innexin 9 and 11 had transcript variants: *Limax* innexin 9x1-9x4 and *Limax* innexin 11x1-11x2. The boxes show the four predicted transmembrane domains (TM1 to TM4), while the red arrows indicate two extracellular loops (EC1 and EC2). The black asterisks indicate the extracellular cysteine residues in the extracellular loops, while the red asterisk indicates the additional cysteine residue. The black arrow-heads indicate the P-X-X-X-W motif.

The deduced protein sequences were used for predicting transmembrane domain using OCTOPUS software [38]. Amino acid alignment analysis revealed that the *Limax* innexin homologs conserve the typical characteristics of innexin proteins, four transmembrane domains that are connected by the first and second extracellular loops and one intracellular loop (Fig 1). Around the second transmembrane domain, each *Limax* innexin had a P-X-X-X-W motif (e.g. *Limax* innexin 1, PNIFW_120-124_; Fig 1) [39]. This motif is conserved in innexin and pannexin protein families and the proline residue functions as a molecular hinge for voltage-dependent gating of connexin channels [40, 41].

Another typical characteristic of innexin proteins is the conserved cysteine (Cys) residues in the extracellular loops [39]. In invertebrate innexin, two pairs of Cys form essential intramolecular disulfide bonds between the first and second extracellular loops [42]. The *Limax* innexin homologs had two Cys residues each in the first and second extracellular loops (Fig 1; e.g. *Limax* innexin 1, Cys_56_ and Cys_74_ in the first extracellular loop; Cys_262_ and Cys_279_ in the second extracellular loop).

Interestingly, *Limax* innexin 1–9, except *Limax* innexin 4, had an additional Cys between the two Cys residues in the first extracellular loop (Fig 1; e.g. *Limax* innexin 1, Cys_58_). The additional Cys residue was also detected in the same region of other innexin and pannexin sequences of limited species reported in the public databases. In invertebrate innexins, the additional Cys residue was detected in six of the 12 leech innexins (*Hirudo medicinalis* innexins 1, 3, 6, 9, 11, and 12) but not found in the innexin sequences of insects and nematodes. In vertebrates, the additional Cys residue was detected in pannexin 3 of the clade *Cetartiodactyla*, such as cattle (*Bos taurus* NP001137556.1) and long-finned pilot whales (*Globicephala melas* XP030691269.1), but not in other pannexin isoforms (pannexin 1 and 2) or in connexins. The function of this additional Cys residue has not been reported in any animal species, however, these results indicate that the additional Cys residue is not a unique feature of molluscan innexins.

Sequence alignment analysis also revealed differences among the identified *Limax* innexins (Fig 1). The lengths of the extracellular loops were well conserved among *Limax* innexin 1–10 (the first extracellular loops, 53–55 amino acid residues; the second extracellular loop, 66–68 amino acid residues). On the other hand, the first extracellular loop in *Limax* innexin 11 was longer than that in *Limax* innexin 1–10 (the first extracellular loop, 69 amino acids; the second extracellular loop, 63 amino acids). This result is similar to the observation in vertebrate connexin and pannexin with different lengths of the extracellular loops [43]. Furthermore, the lengths between two Cys residues in the second extracellular loops largely differed between *Limax* innexin 1–10 and *Limax* innexin 11 (*Limax* innexin 1–10, 16 amino acids; *Limax* innexin 11, 12 amino acids). Previous studies on vertebrate connexins have reported that the amino acid sequences between the Cys are critical for the formation of functional intercellular channels [9, 44, 45]. By focusing on the differences between vertebrate connexins and pannexins, the isoforms of *Limax* innexin can be broadly divided into two groups, *Limax* innexin 1–10 and *Limax* innexin 11.

### Molecular phylogenetic analysis of molluscan innexin homologs

**Fig 2:**
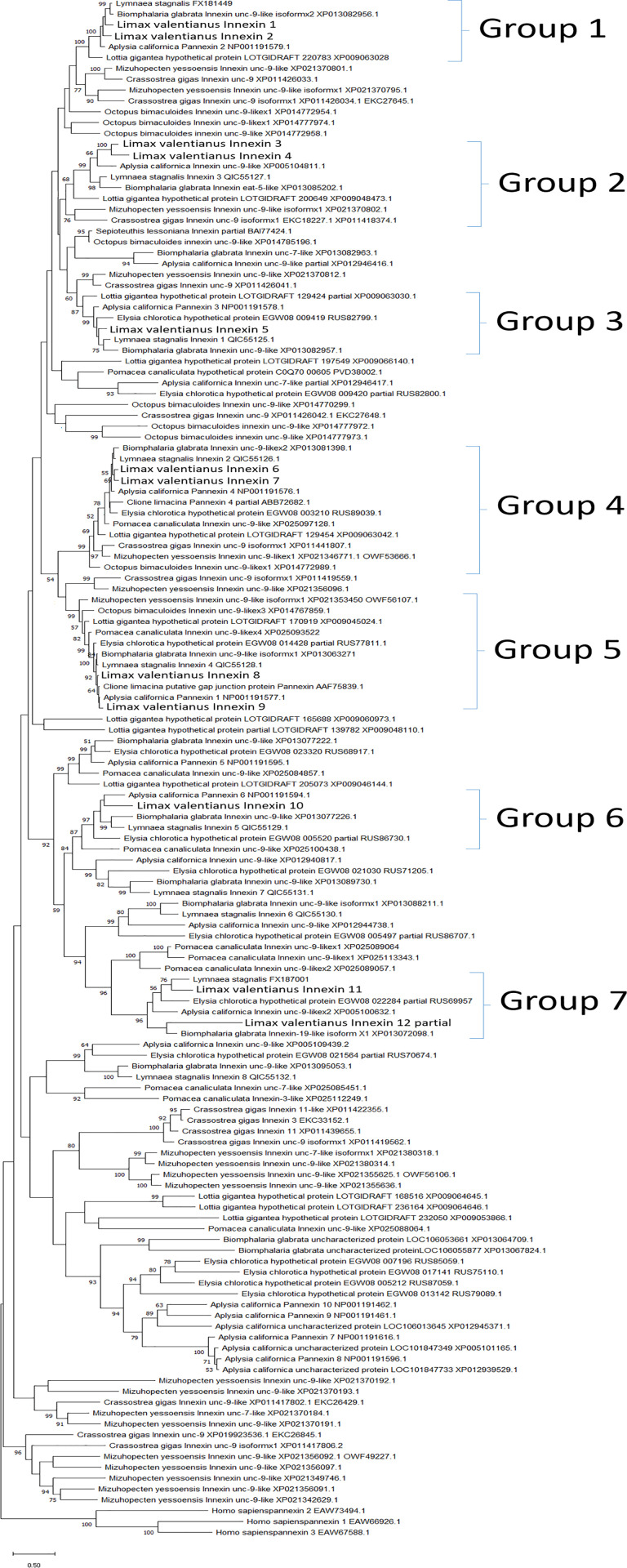
Phylogenetic tree of molluscan innexins. Phylogenetic analysis of molluscan innexins was performed using the maximum likelihood method with *Homo sapiens* pannexins as the outgroup. The percentage of replicate trees in which the associated taxa clustered together in the bootstrap test (2000 replicates) is shown next to the branches (only values greater than 50 are displayed). Sequence accession numbers are indicated next to species names. The names of *Limax* innexin homologs are in bold font.

Next, molecular phylogenetic analysis was performed to classify and examine the characteristics of the identified *Limax* innexins. The innexin genes exhibited phylum-specific diversification in each phylum Arthropoda, Nematoda, and Mollusca [46]. Therefore, we performed the phylogenetic analysis using available molluscan innexin sequences in the Swissprot, Genbank, and Refseq databases. Additionally, the hypothetical protein sequences from the genomic data of *Elysia chlorotica* (BioProject PRJNA484060; sequence and assembled genome) and previously reported molluscan innexin sequences were used [26, 27]. In the pond snail *Lymnaea stagnalis*, in addition to the reported eight innexin transcripts (LstInx1 to LstInx8) [27], we further identified two homolog sequences in other transcriptome data of the CNS (*Lymnaea* FX186689 and FX187001) [29]. When splicing variants were found, one variant of each gene was used for the analysis. Because the mammalian pannexins are homologous to the invertebrate innexins [47], we here used *Homo sapiens* pannexins as the outgroup. The accession numbers for the same amino acid sequence are summarized in Fig 2 (e.g. *Crassostrea gigas* unc-9 isoformx1: EKC18227.1, XP011418374.1). The numbers of sequences in different animal species were as follows: *n* = 20 for *Aplysia californica* (Gastropoda); *n* = 10 for *Lymnaea stagnalis* (Gastropoda); *n* = 2 for *Clione limacina* (Gastropoda); *n* = 14 for *Biomphalaria glabrata* (Gastropoda); *n* = 11 for *Pomacea canaliculata* (Gastropoda); *n* = 12 for *Lottia gigantea* (Gastropoda); *n* = 14 for *Elysia chlorotica* (Gastropoda); *n* = 20 for *Mizuhopecten yessoensis* (Bivalvia); *n* = 14 for *Crassostrea gigas* (Bivalvia); *n* = 9 for *Octopus bimaculoides* (Cephalopoda); n = 1 for *Sepioteuthis lessoniana* (Cephalopoda).

Even though the genomic data is available in *Aplysia* and *Elysia*, the number of innexin homolog genes varied among species (*n* = 20 for *Aplysia californica*; *n* = 14 for *Elysia chlorotica*). Consistent with this observation, a previous study on vertebrate connexins demonstrated that the number of connexin genes in zebrafish (n = 37) was approximately two times higher than that in humans (n = 20) and mice (n = 19) [48]. The authors suggested that gene duplication events occur continuously in each phylum, which contributes to the species-specific regulation by gap junction-related proteins.

Molecular phylogenetic analysis revealed that the identified *Limax* innexin gene transcripts expressed in the CNS can be classified into seven ortholog groups (Fig 2; Group 1, *Limax* innexin 1 and 2; Group 2, *Limax* innexin 3 and 4; Group 3, *Limax* innexin 5; Group 4, *Limax* innexin 6 and 7; Group 5, *Limax* innexin 8 and 9; Group 6, *Limax* innexin 10; Group 7, *Limax* innexin 11 and 12).

### Prediction of post-translational modification sites: N-glycosylation, S-nitrosylation, and phosphorylation

**Fig 3:**
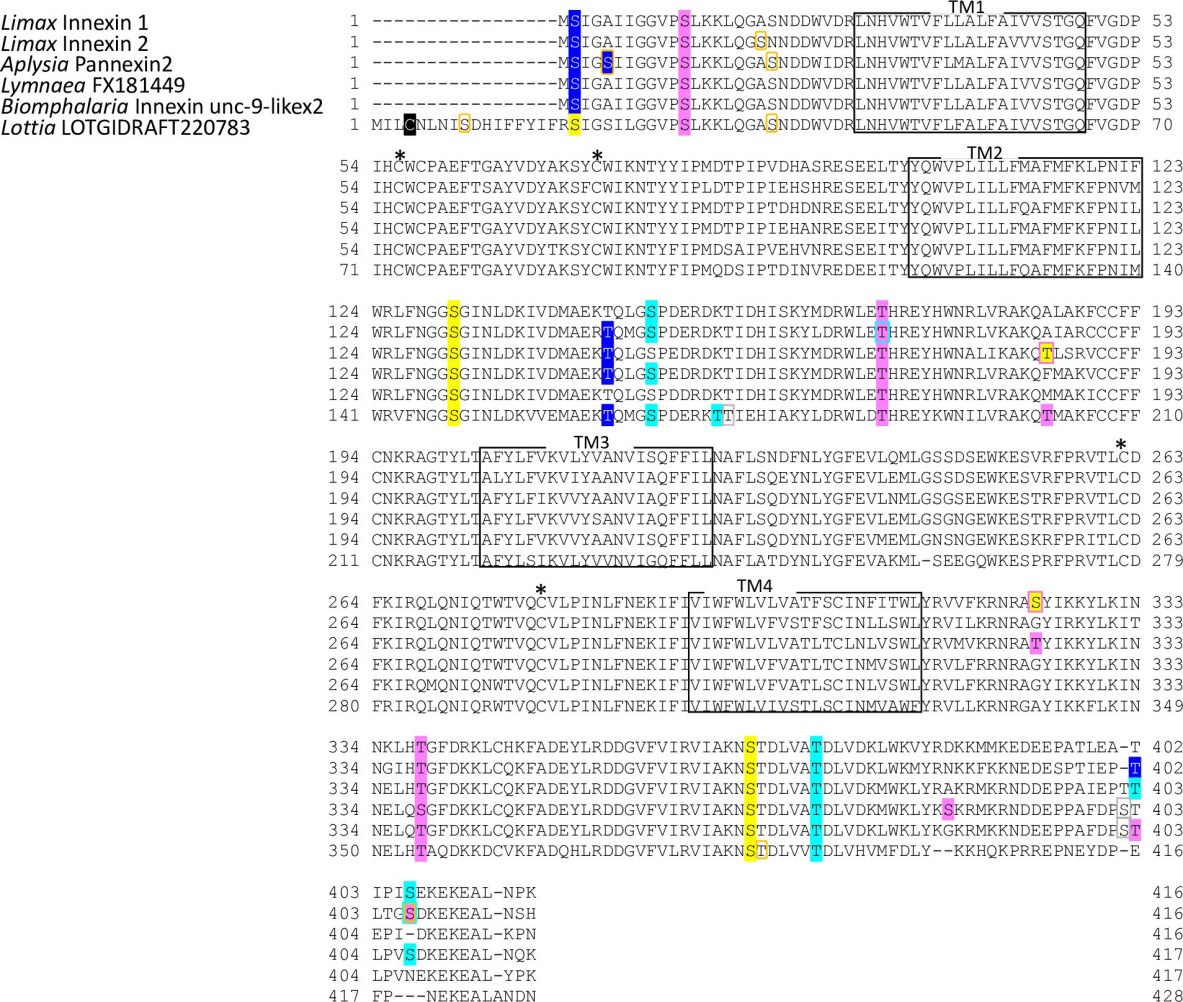
Protein sequence alignment of molluscan innexin orthologs in Group 1. Alignment of deduced amino acid sequences of *Limax* innexin 1 and 2 with orthologous innexin sequences. The boxes show the four transmembrane domains, while the asterisks indicate the extracellular cysteine residues. The potential S-nitrosylation sites are shaded in black. The phosphorylation sites are indicated with shading or box in magenta (protein kinase C), yellow (protein kinase A), blue (casein kinase 1), cyan (casein kinase 2), orange (P34cdc), and gray (protein kinase G).

**Fig 4:**
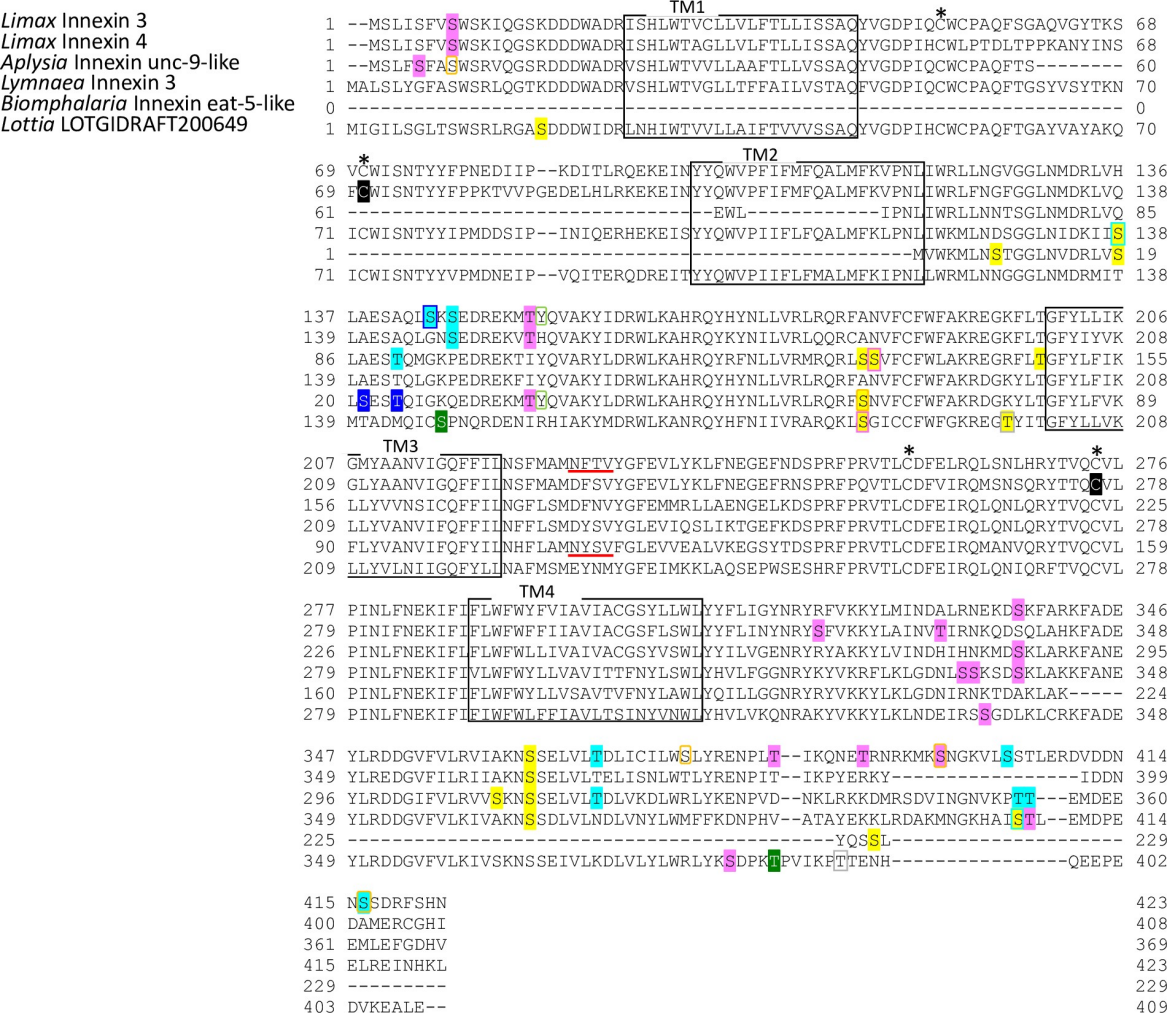
Protein sequence alignment of molluscan innexin orthologs in Group 2. Alignment of deduced amino acid sequences of *Limax* innexin 3 and 4 with orthologous innexin sequences. The boxes show the four transmembrane domains, while the asterisks indicate the extracellular cysteine residues. The potential S-nitrosylation sites are shaded in black. The phosphorylation sites are indicated with shading or box in magenta (protein kinase C), yellow (protein kinase A), blue (casein kinase 1), cyan (casein kinase 2), orange (P34cdc2), green (p38-mitogen-activated protein kinase), light green (proto-oncogene tyrosine-protein kinase Src), and gray (protein kinase G). Potential N-glycosylation sites are underlined with bold red lines.

**Fig 5:**
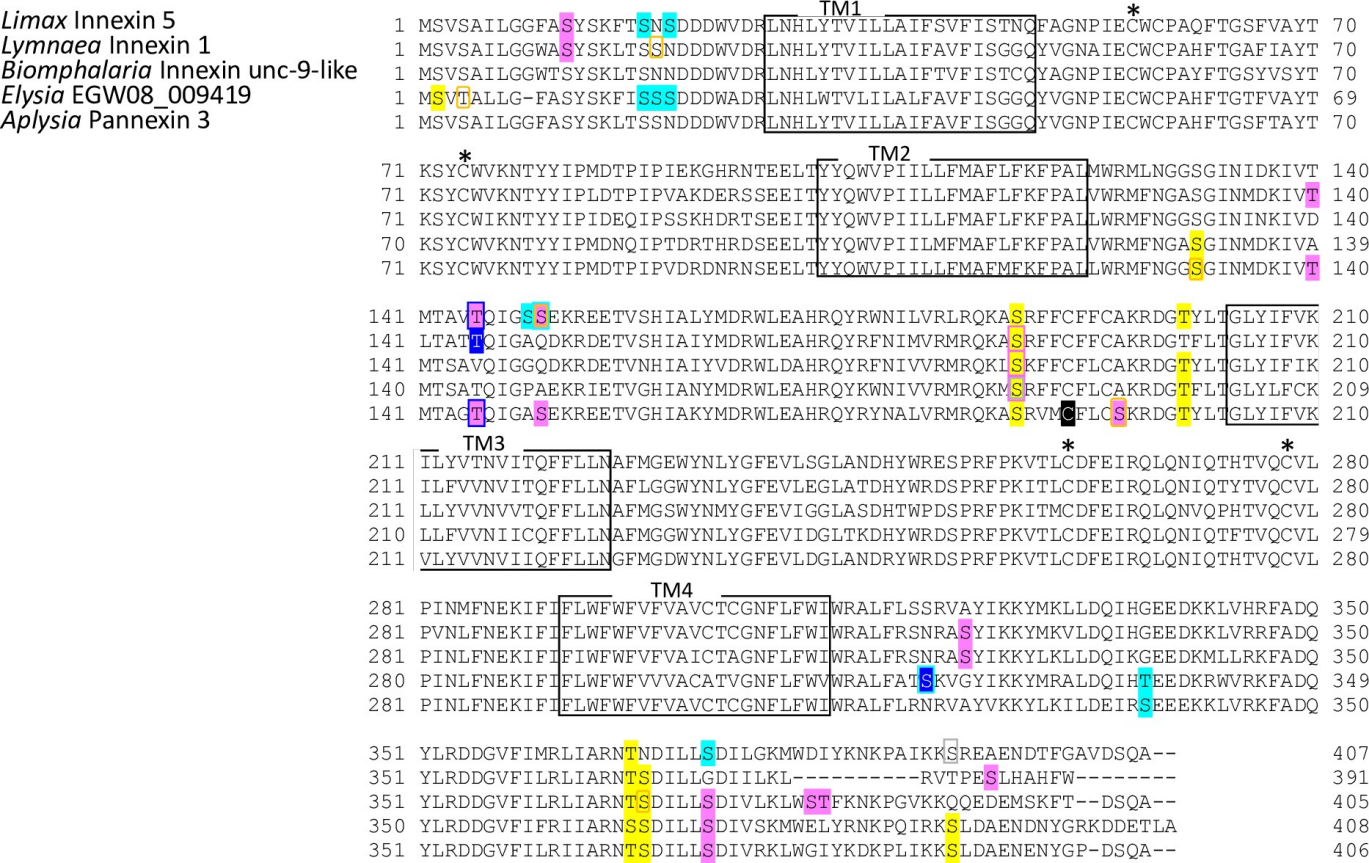
Protein sequence alignment of molluscan innexin orthologs in Group 3. Alignment of deduced amino acid sequences of *Limax* innexin 5 with orthologous innexin sequences. The boxes show the four transmembrane domains, while the asterisks indicate the extracellular cysteine residues. The potential S-nitrosylation sites are shaded in black. The phosphorylation sites are indicated with shading or box in magenta (protein kinase C), yellow (protein kinase A), blue (casein kinase 1), cyan (casein kinase 2), orange (P34cdc2), and gray (protein kinase G).

**Fig 6:**
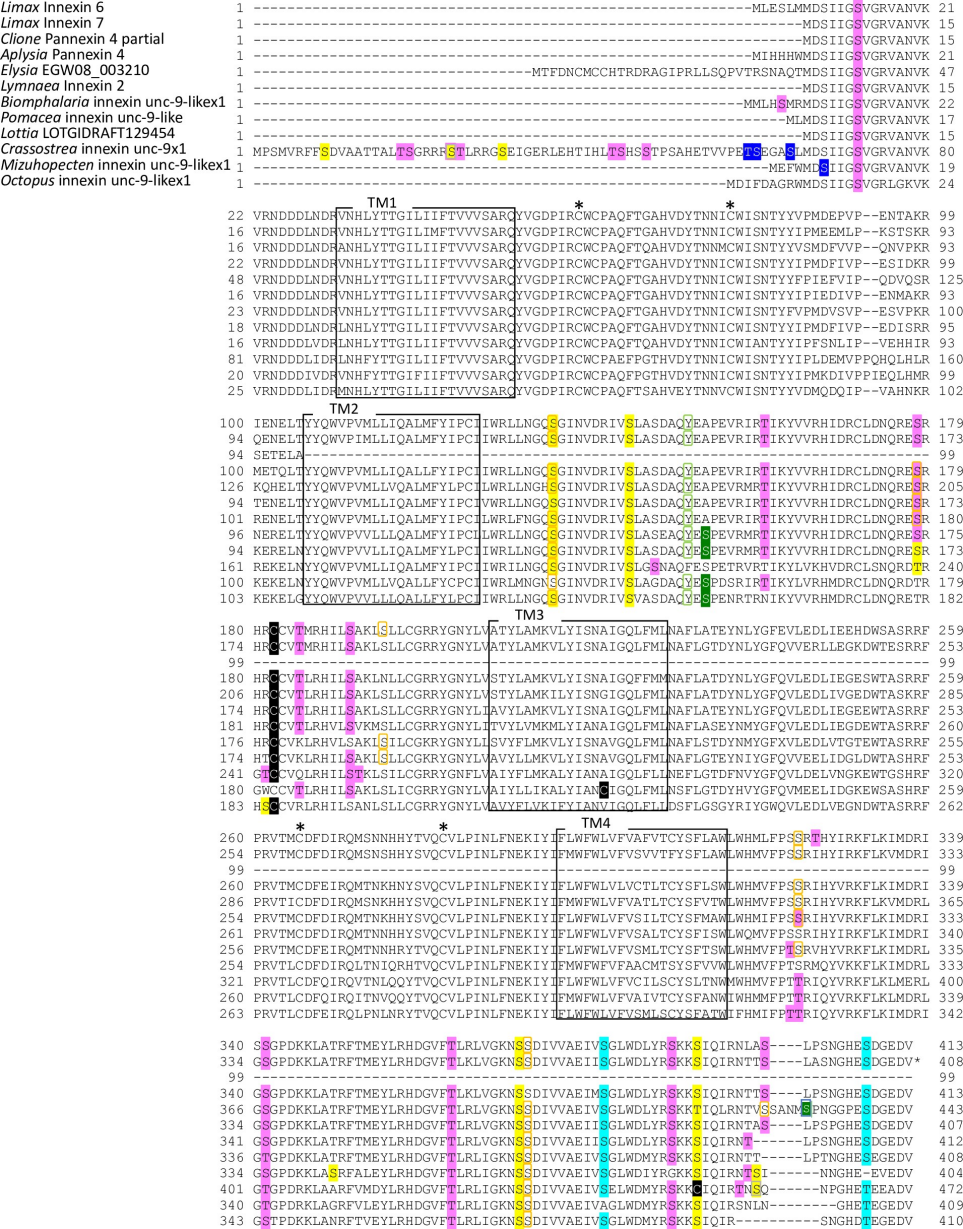
Protein sequence alignment of molluscan innexin orthologs in Group 4. Alignment of deduced amino acid sequences of *Limax* innexin 6 and 7 with orthologous innexin sequences. The boxes show the four transmembrane domains, while the asterisks indicate the extracellular cysteine residues. The potential S-nitrosylation sites are shaded in black. The phosphorylation sites are indicated with shading or box in magenta (protein kinase C), yellow (protein kinase A), blue (casein kinase 1), cyan (casein kinase 2), orange (P34cdc2), green (p38-mitogen-activated protein kinase), light green (proto-oncogene tyrosine-protein kinase Src), and gray (protein kinase G).

**Fig 7:**
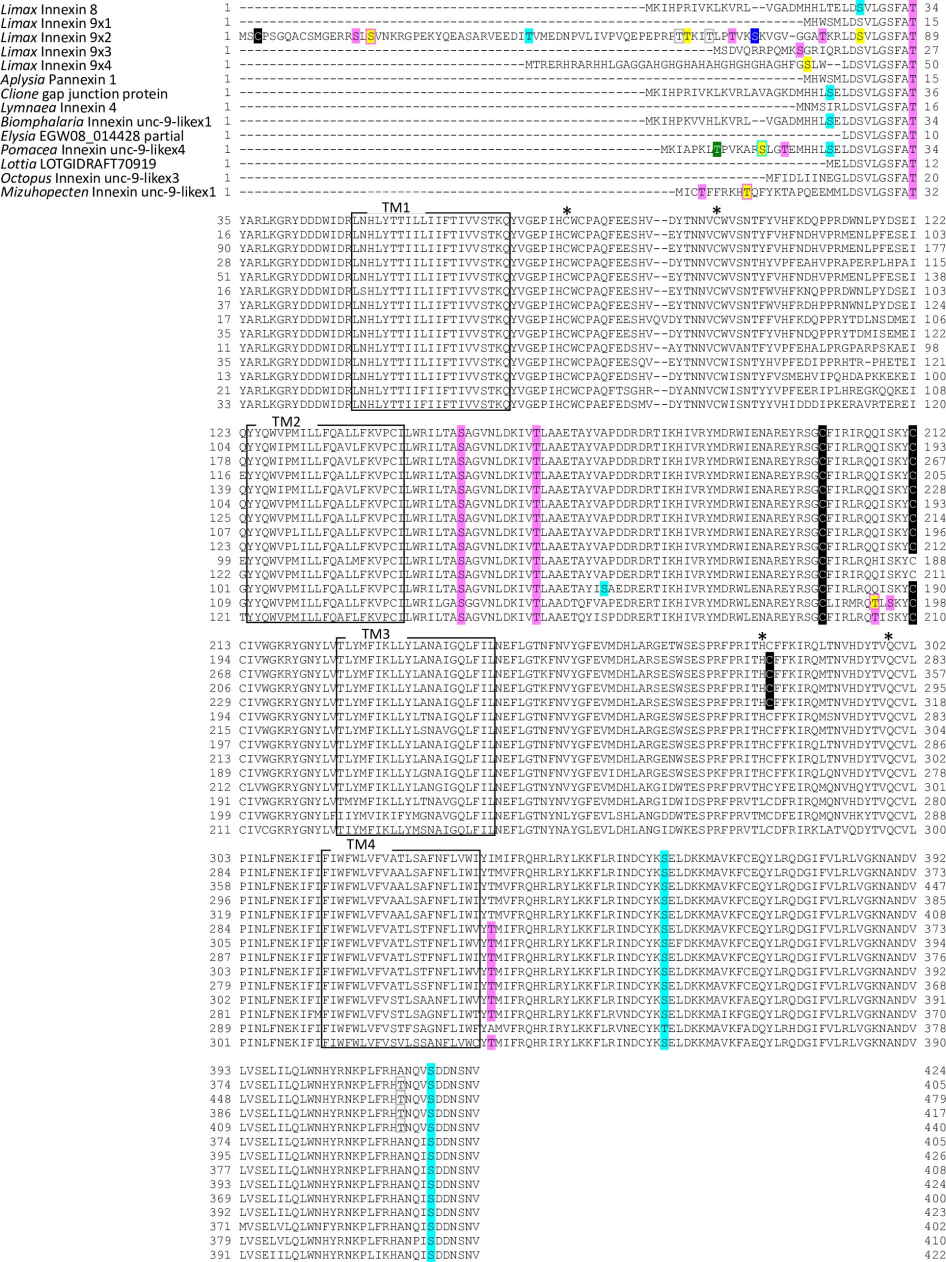
Protein sequence alignment of molluscan innexin orthologs in Group 5. Alignment of deduced amino acid sequences of *Limax* innexin 8 and 9 with orthologous innexin sequences. The boxes show the four transmembrane domains, while the asterisks indicate the extracellular cysteine residues. The potential S-nitrosylation sites are shaded in black. The phosphorylation sites are indicated with shading or box in magenta (protein kinase C), yellow (protein kinase A), blue (casein kinase 1), cyan (casein kinase 2), green (p38-mitogen-activated protein kinase), and gray (protein kinase G). Potential N-glycosylation sites are underlined with bold red lines.

**Fig 8:**
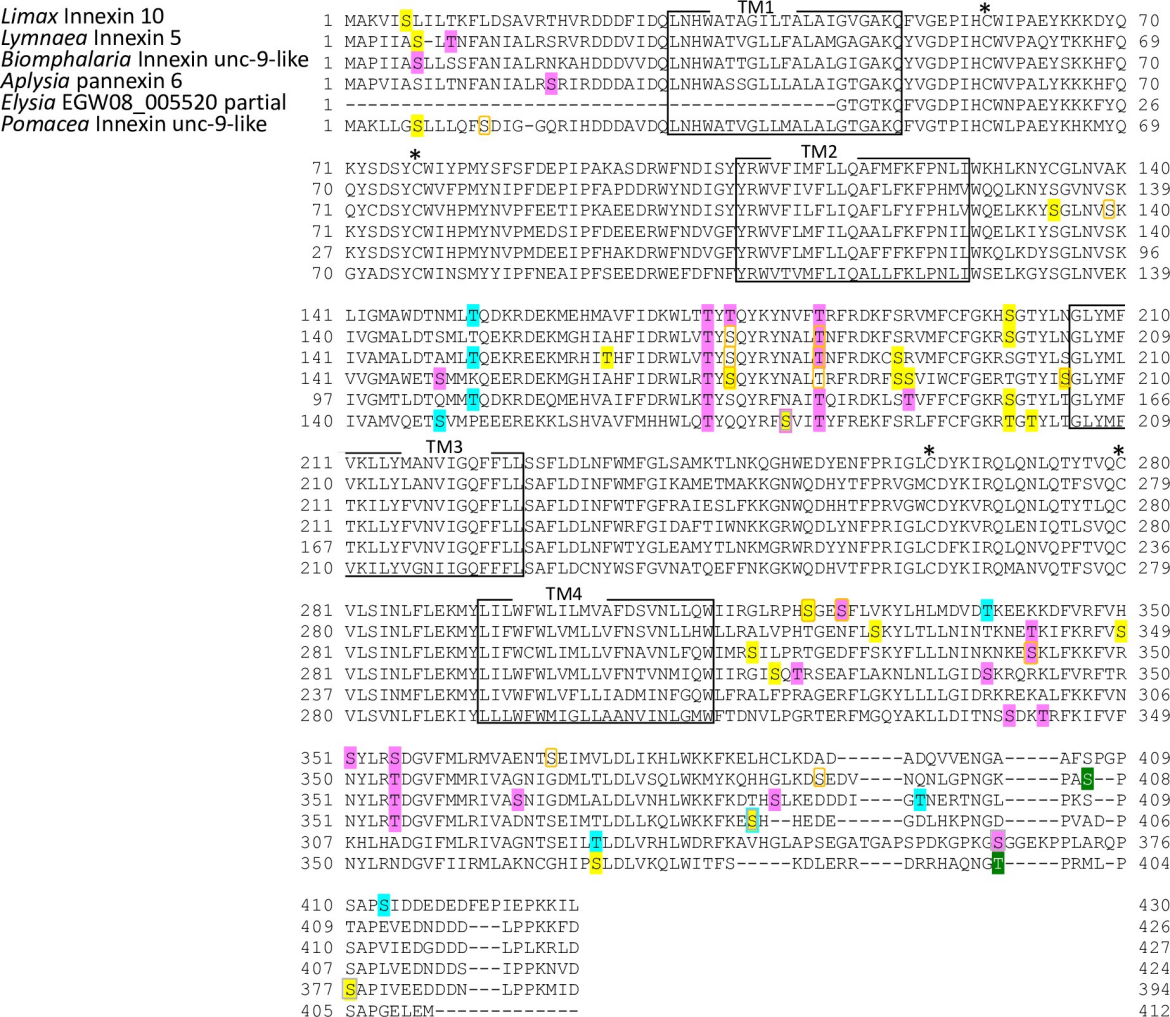
Protein sequence alignment of molluscan innexin orthologs in Group 6. Alignment of deduced amino acid sequences of *Limax* innexin 10 with orthologous innexin sequences. The boxes show the four transmembrane domains, while the asterisks indicate the extracellular cysteine residues. The phosphorylation sites are indicated with shading or box in magenta (protein kinase C), yellow (protein kinase A), cyan (casein kinase 2), orange (P34cdc2), green (p38-mitogen-activated protein kinase), and gray (protein kinase G).

**Fig 9:**
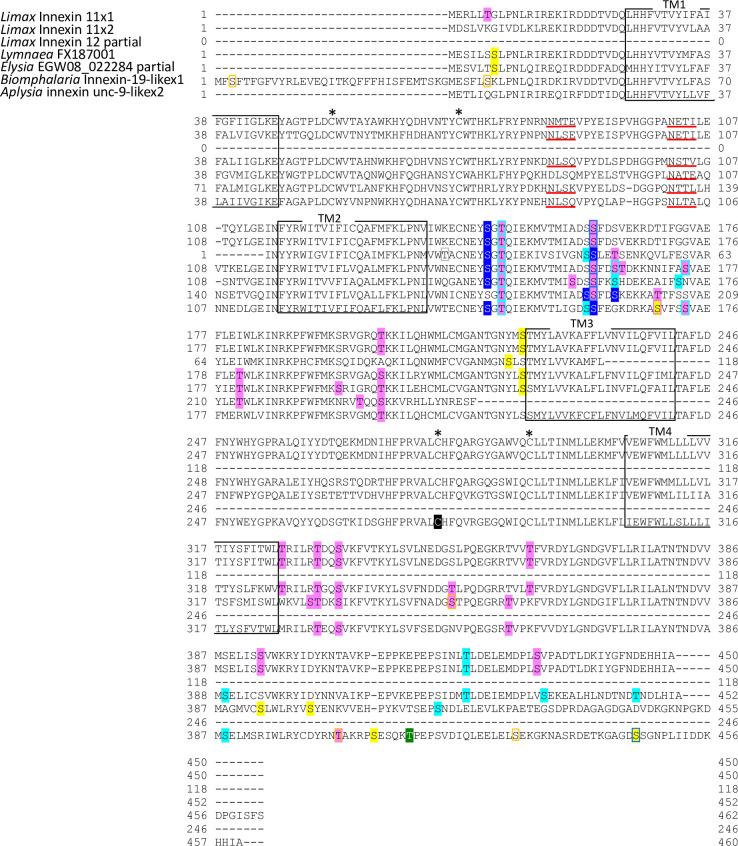
Protein sequence alignment of molluscan innexin orthologs in Group 7. Alignment of deduced amino acid sequences of *Limax* innexin 11 and 12 with orthologous innexin sequences. The boxes show the four transmembrane domains, while the asterisks indicate the extracellular cysteine residues. The potential S-nitrosylation sites are shaded in black. The phosphorylation sites are indicated with shading or box in magenta (protein kinase C), yellow (protein kinase A), blue (casein kinase 1), cyan (casein kinase 2), orange (P34cdc2), green (p38-mitogen-activated protein kinase), and gray (protein kinase G). Potential N-glycosylation sites are underlined with bold red lines.

**Fig 10:**
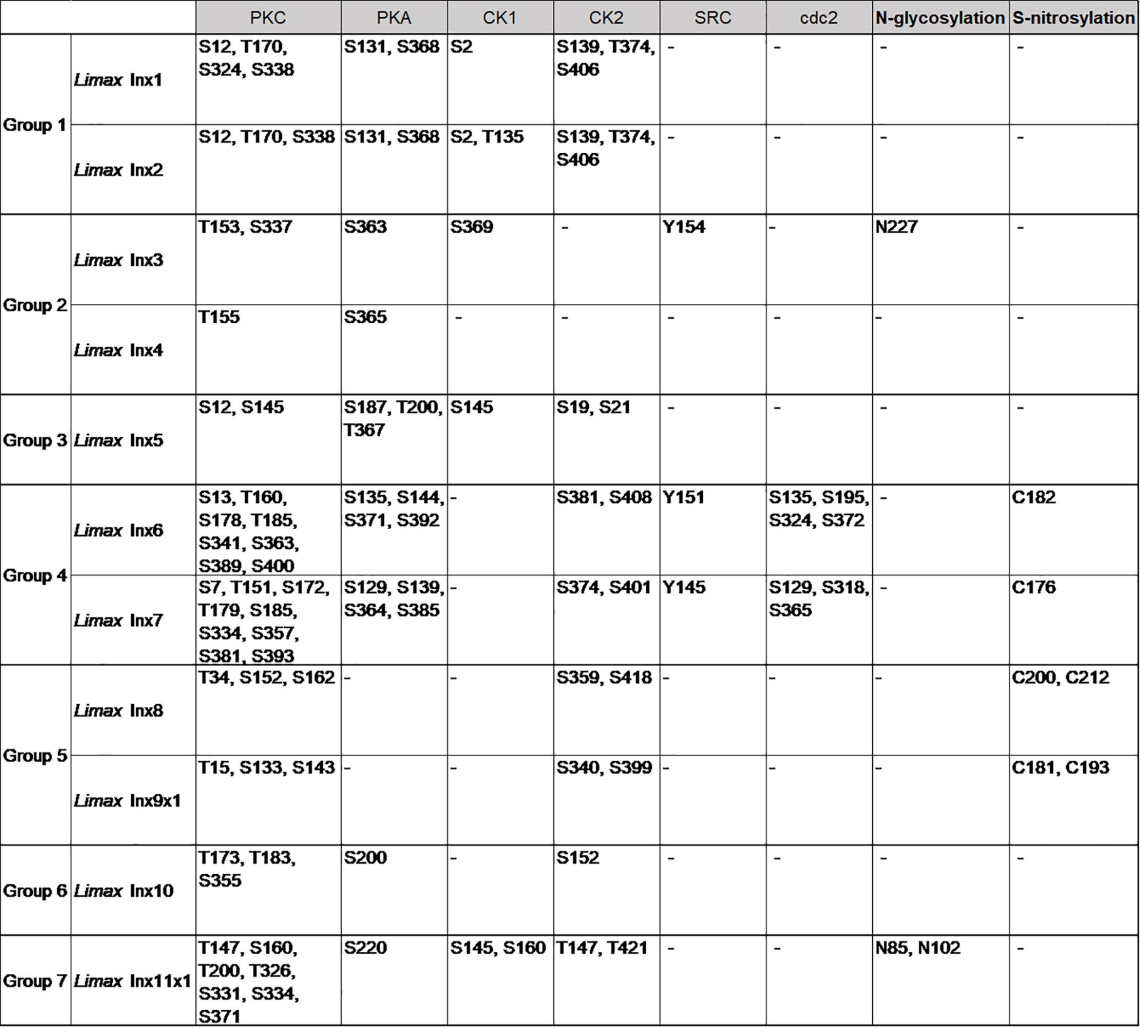
Summary of the predicted modification sites in the innexin orthologs of the seven groups. The conserved modification sites between *Limax* innexin sequence and at least one other ortholog sequence are shown.

To classify the molluscan innexin homologs, potential protein modification sites were detected in the deduced amino acid sequences of the seven ortholog groups. The predicted modification sites in the innexin sequences of each group are shown in Figs 3–9. The conserved modification sites between orthologs are summarized in Fig 10.

Potential N-glycosylation sites were detected only in the innexin homologs of Group 2 and 7. In Group 2, one potential N-glycosylation site was identified at the first extracellular loop in two of the six orthologs (*Limax* innexin 3, N_227_FT; *Biomphalaria* XP013085202.1 partial CDS sequence, N_110_YS). In Group 7, two N-glycosylation sites were identified in the first extracellular loop in all orthologs of Group 7 (*Limax* innexin 11 splice variants, *Limax* innexin 11x1, N_85_MT and N_102_ET; *Limax* innexin 11x2, N_85_LS and N_102_ET; *Lymnaea* FX187001, N_85_LS and N_102_ET; *Elysia* EGW08_022284 partial sequence, N_102_AT; *Biomphalaria* innexin-19-likex1 N-terminal partial sequence, N_118_LS and N_134_TT; *Aplysia* innexin unc-9-likex2, N_85_LS and N_101_LT), except *Limax* innexin 12 for which we did not obtain the full-length coding sequence.

The result revealed that N-glycosylation modification occurs in the molluscan innexins of certain ortholog groups. Consistent with this finding, vertebrate pannexin proteins have N-glycosylation sites at the extracellular loops, whereas nearly all connexins do not have the N-glycosylation sites. N-glycosylation of pannexins regulates the subcellular localization and intermixing of different pannexins for channel formation [49, 50]. Additionally, N-glycosylation of pannexin inhibits the intercellular docking of neighboring pannexin channels and the formation of gap junctions [50, 51]. To summarize the results so far, molluscan innexins of Group 7 exhibited the following two pannexin-like characteristics: N-glycosylation at the extracellular loops and longer extracellular loops compared to other homologs in Group 1–6.

We also identified S-nitrosylation sites in the molluscan innexin homologs of the seven groups. As a result, the conserved S-nitrosylation sites were found in the intracellular loops of limited ortholog groups, Group 4 and 5 (Figs 6, 7 and 10). S-nitrosylation, which is the covalent addition of nitric oxide (NO) to the Cys residue, is reported to be involved in the regulation of channel properties, such as channel permeability and voltage-sensitive gating of vertebrate connexins and pannexins [52–54]. In molluscs, various studies have examined NO function in the CNS [24, 55–60]. Our findings gave a new insight that NO regulates neuronal communication in molluscan CNS by modifying the gap junction-related proteins.

Further, we identified the phosphorylation sites of multiple kinases, PKC, PKA, PKG, CK1, CK2, SRC, cdc2, CaMK, and p38MAPK (Figs 3–10). These kinases regulate the protein functions of vertebrate connexin/pannexin, such as oligomerization, channel properties, intracellular trafficking, gap junction assembly, and stability [5, 6, 61, 62]. The results showed that the phosphorylation sites for PKC, PKA, CK1, CK2, SRC and cdc2 were relatively conserved among the orthologs in the same group (Fig 10). The phosphorylation sites for PKG, CaMK, and p38MAPK were not conserved in the ortholog groups. Although Group 4 and 5 were phylogenetically related (Fig 2), a surprisingly high number of phosphorylation sites was found in the orthologs in Group 4 compared with Group 5 (Figs 6, 7 and 10). The results showed that the protein kinase phosphorylation sites are conserved among the orthologs and that each ortholog group exhibits a distinctive phosphorylation pattern.

Exceptionally, the orthologs of Group 2 exhibited low sequence homology and variability of the protein modification sites (Figs 4 and 10). More, although *Limax* innexin 3 and 4 of Group 2 showed the highest sequence similarity, the protein modification sites were largely different between them (Figs 2, 4 and 10). At the extracellular loop, *Limax* innexin 3 had an N-glycosylation site, while *Limax* innexin 4 had two S-nitrosylation sites. We identified extra phosphorylation sites in the intracellular C-terminal domain of *Limax* innexin 3 compared to *Limax* innexin 4 (Fig 4; T_385_, T_391_, and S_398_ for PKC; T_369_, S_405_, and S_416_ for CK2; S_377_ and S_398_ for cdc2). Similar to this finding, the numbers of phosphorylation sites in the intracellular C-termini show large variability among vertebrate connexin isoforms [61]. For instance, connexin 43 is phosphorylated by at least five different protein kinases at more than 12 motifs, while connexin 26 has no phosphorylation motif in this region. The phosphorylation of connexin C-terminus has been reported to regulate the trafficking, assembly, and degradation [61]. Thus, *Limax* innexin 3 and 4 channels may be differently regulated by phosphorylation, even though they are phylogenetically close to each other.

### CircRNA identification of *Limax* innexin genes

CircRNA is a class of non-coding RNAs that are generated through alternative back-splicing, which connects the terminal 5’ and 3’ ends of linear pre-mRNA [63], and the function has not been fully elucidated. Previous studies have demonstrated the presence of circRNAs throughout animal species, such as mammals, insects, and nematodes [64, 65]. In the brain tissue, circRNA is especially abundant and lots of circRNA-producing genes encode synapse-related proteins [64, 66].

In the RT-PCR experiments of *Limax* innexin transcripts in the CNS, we obtained multiple amplicons for some innexin homologs. Sequencing analysis revealed that these amplicons were circRNAs. To identify the circRNAs of the innexin genes, we designed divergent primers for the coding region of *Limax* innexin sequences, as a previous study showed that circRNAs mainly comprise coding exons [65]. As a result, we identified 16 circRNA sequences for nine *Limax* innexin genes in the CNS (Table 1). Nine circRNAs each encoded a truncated protein lacking the N-terminal end (circINX2b, circINX2c, circINX4, circINX6b, circINX7a, circINX8a, circINX8b, circINX9x2, and circINX9x4), and three circRNAs each encoded the full-length protein (circINX2a, circINX3, and circINX7b).

**Table 1:** Summary of identified *Limax* innexin circular RNAs (circRNAs) in the central nervous system.

circRNA name	length	Start-end position in the CDS (total length of the CDS)	Region out of the CDS
circINX2a	*1395*	1–1251 (1251)	144
circINX2b	*801*	282–1251 (1251)	0
circINX2c	*1027*	447–1251 (1251)	222
circINX3	*1504*	1–1269 (1269)	235
circINX4	*1965*	215–1227 (1227)	952
circINX5	*1192*	1–1162 (1224)	30
circINX6a	*633*	458–1090 (1242)	0
circINX6b	*1246*	54–1242 (1242)	57
circINX7a	*879*	535–1224 (1224)	367
circINX7b	*1235*	1–1224 (1224)	11
circINX8a	*1057*	223–1275 (1275)	4
circINX8b	*1472*	73–1275 (1275)	269
circINX9x2	*3234*	223–1440 (1440)	2016
circINX9x4	*1410*	26–1323 (1323)	112
circINX11x1a	*795*	265–1059 (1353)	0
circINX11x1b	*784*	312–1095 (1353)	0

CDS, coding domain sequence.

The functions of circRNAs generated from the connexin, pannexin, and innexin genes have not been elucidated. Thus, we also examined the circRNAs of vertebrate connexin and pannexin genes using a database that contains >32,000 human circRNAs [67], and retrieved circRNA data of four connexin genes (GJA1, connexin 43; GJB3, connexin 31; GJB5, connexin 31.1; GJC1, connexin 45) and one pannexin gene (PNX1, pannexin 1). Of these, circRNAs of connexin 45 (hsa_circ_0106948, hsa_circ_0106949, hsa_circ_0106950, hsa_circ_0106951, hsa_circ_0106952) and pannexin 1 (hsa_circ_0096811, hsa_circ_0096812, hsa_circ_0096813, and hsa_circ_0096814) were listed as the transcripts in the brain tissue.

The results demonstrated that circRNAs are constitutively formed from the innexin genes in the CNS of *Limax*, and that circRNA formation from gap junction-related protein genes is not a characteristic event in molluscs. The observation suggested that circRNAs can be involved in the regulatory mechanism of certain gap junction-related proteins.

## Discussion

In this study, the innexin homologs expressed in the CNS of *Limax* were identified and characterized. Phylogenetic analysis demonstrated the diversity of molluscan innexin genes and the ortholog groups. The prediction of post-translational modifications, including phosphorylation, S-nitrosylation, and N-glycosylation, further revealed the characteristics of each ortholog group. Additionally, we identified the abundant expression of circRNA of innexin homologs in the CNS.

In invertebrate species, innexin is the only protein family that forms gap junctions. Even though innexin sequence data in molluscan species have been accumulated through comprehensive sequence analysis studies, the multiple isoforms have not been classified. Based on the phylogenetic analysis, we classified *Limax* innexin homologs into seven molluscan ortholog groups. One ortholog group, Group 7, exhibited vertebrate pannexin-like characteristics at the extracellular loops with the lengths and the glycosylation sites. In vertebrates, previous studies have demonstrated that the intercellular docking of two single-cell membrane channels is regulated by the interaction between the extracellular loops of gap junction-related proteins. Pannexin proteins mainly form single-cell membrane channels *in vivo*, and glycosylation of pannexin at the extracellular loop inhibits the formation of intercellular channels [12, 51]. In invertebrates, only one study examined the glycosylation of innexin homologs in the yellow fever mosquito *Aedes aegypti* [68]. The study reported that two of the six *Aedes* innexins (AeInx3 and AeInx7) have putative glycosylation sites and that AeInx3 is an important component of gap junctions. However, AeInx3 exhibited multiple bands in the western blot, that indicates several stages of modifications on AeInx3. Regard to the previous studies of pannexin, the glycosylation status of AeInx3 protein can inhibit the formation of intercellular gap junction channels, and the authors mentioned the effect of glycosylation on intercellular channel formation. In our study, in addition to the pannexin-like orthologs in Group 7, *Limax* innexin 3 in Group 2 had an N-glycosylation site. This indicates that the N-glycosylation status of *Limax* innexin 3 possibly regulates the intercellular docking of the channels.

In addition to the N-glycosylation sites, we identified the phosphorylation and S-nitrosylation sites and characterized molluscan innexins of the seven ortholog groups. Each group exhibited a distinctive protein modification pattern and the protein modification sites were well conserved among the orthologs (Fig 10). The results indicate that the regulatory mechanism of innexin channels is well conserved among orthologs and the duplication of innexin genes have generated innexin isoforms with new functions, as previously reported for vertebrate connexins [48]. Exceptionally, the orthologs in Group 2 exhibited low sequence homology and did not conserve N-glycosylation sites and protein modification sites (Figs 2 and 10). The variability among the orthologs in Group 2 may arise from continuous gene duplication events that produce new functions of innexin proteins in Group 2.

Further, we examined the formation of circRNAs from *Limax* innexin genes. We also identified circRNAs of human connexin and pannexin in the circRNA database [67]. Even though a lot of research has been conducted on gap junction-related proteins in vertebrates, there is no study that examined the existence of circRNA. So far, only one review proposed that mammalian connexin 43-derived circRNA functions as a miRNA sponge during breast cancer initiation stages [69], however, this has not been experimentally demonstrated yet. The function of circRNAs has been under investigation and several studies reported that circRNAs function as miRNA sponges [65, 70]. On the other hand, recent studies further reported that several circRNAs are associated with polysomes and translated [71, 72]. In this study, we identified twelve of the 16 *Limax* innexin circRNAs encoding N-terminal truncated innexin proteins, indicating that circRNAs can be templates for translation. Interestingly, several studies on vertebrate connexin reported the unexpected functions of N-terminal truncated connexin that are generated from internal translation initiation [73–75]. The short connexin 43 isoform is involved in trafficking the full-length connexin 43 to the gap junction site. Additionally, the short connexin 43 isoform directly regulates N-cadherin transcription, which results in neural crest cell migration at an early developmental stage in amphibian and mammalian cells. Thus, the translation of *Limax* innexin circRNA may generate truncated innexin proteins that have uncharacterized roles, such as protein trafficking and regulation of gene expression.

As described above, there are multiple innexin gene products, including circRNAs, expressed in the CNS of *Limax*. The present data directly contribute to future analysis of gene expression using quantitative RT-PCR or *in situ* hybridization in the CNS. In order to elucidate the physiological functions of each innexin homologs on electrical synapses, such as local electrical junctions of bursting neurons in the *Limax* procerebral lobe [23, 24], it would be helpful to apply gene knockout systems using siRNA (short interfering RNA) and CRISPR/CAS9 systems to electrophysiology experiments.

## Conclusion

In this study, multiple innexin transcripts were identified in the CNS of *Limax* and the molluscan innexin sequences were phylogenetically classified. Additionally, circRNA formation of multiple innexin genes was demonstrated, which provided novel insights for understanding the regulatory mechanism of gap-junction-related proteins. The results of this study will facilitate further research on the function of innexin proteins and contribute to the development of new research on gap junction proteins.

## Supporting information

S1 Table PCR primers used in this study. (DOCX)

## References

[1] Welzel G, Schuster S. Long-term potentiation in an innexin-based electrical synapse. Sci Rep [Internet]. 2018;8(1):12579. Available from: 10.1038/s41598-018-30966-w 30135467 PMC6105662

[2] Bhattacharya A, Aghayeva U, Berghoff EG, Hobert O. Plasticity of the Electrical Connectome of C. elegans. Cell. 2019;176(5):1174–1189.e16. 10.1016/j.cell.2018.12.024 30686580 PMC10064801

[3] Zoidl G, Petrasch-Parwez E, Ray A, Meier C, Bunse S, Habbes HW, et al. Localization of the pannexin1 protein at postsynaptic sites in the cerebral cortex and hippocampus. Neuroscience. 2007;146(1):9–16. 10.1016/j.neuroscience.2007.01.061 17379420

[4] D’hondt C, Iyyathurai J, Vinken M, Rogiers V, Leybaert L, Himpens B, et al. Regulation of connexin- and pannexin-based channels by post-translational modifications. Biol Cell. 2013;105(9):373–98. 10.1111/boc.201200096 23718186

[5] Pogoda K, Kameritsch P, Retamal MA, Vega JL. Regulation of gap junction channels and hemichannels by phosphorylation and redox changes: a revision. BMC Cell Biol [Internet]. 2016;17(Suppl 1). Available from: 10.1186/s12860-016-0099-3 PMC4896245 27229925

[6] Boyce AKJ, Epp AL, Nagarajan A, Swayne LA. Transcriptional and post-translational regulation of pannexins. Biochim Biophys Acta—Biomembr [Internet]. 2018;1860(1):72–82. Available from: 10.1016/j.bbamem.2017.03.004 28279657

[7] Beyer EC, Berthoud VM. Gap junction gene and protein families: Connexins, innexins, and pannexins. Biochim Biophys Acta—Biomembr [Internet]. 2018;1860(1):5–8. Available from: 10.1016/j.bbamem.2017.05.016 28559187 PMC5704981

[8] Barbe MT, Monyer H, Bruzzone R. Cell-cell communication beyond connexins: The pannexin channels. Vol. 21, Physiology. 2006. p. 103–14. 10.1152/physiol.00048.2005 16565476

[9] Maeda S, Nakagawa S, Suga M, Yamashita E, Oshima A, Fujiyoshi Y, et al. Structure of the connexin 26 gap junction channel at 3.5 Å resolution. Nature [Internet]. 2009;458(7238):597–602. Available from: 10.1038/nature07869 19340074

[10] Oshima A, Tani K, Fujiyoshi Y. Atomic structure of the innexin-6 gap junction channel determined by cryo-EM. Nat Commun. 2016;7. 10.1038/ncomms13681 27905396 PMC5146279

[11] Penuela S, Gehi R, Laird DW. The biochemistry and function of pannexin channels. Biochim Biophys Acta—Biomembr [Internet]. 2013;1828(1):15–22. Available from: 10.1016/j.bbamem.2012.01.017 22305965

[12] Sosinsky GE, Boassa D, Dermietzel R, Duffy HS, Laird DW, MacVicar BA, et al. Pannexin channels are not gap junction hemichannels. Channels. 2011;5(3):193–7. 10.4161/chan.5.3.15765 21532340 PMC3704572

[13] Rothwell CM, De Hoog E, Spencer GE. The role of retinoic acid in the formation and modulation of invertebrate central synapses. J Neurophysiol. 2017;117(2):692–704. 10.1152/jn.00737.2016 27852736 PMC5292328

[14] Katz PS, Quinlan PD. The importance of identified neurons in gastropod molluscs to neuroscience. Curr Opin Neurobiol [Internet]. 2019;56:1–7. Available from: 10.1016/j.conb.2018.10.009 30390485

[15] Dyakonova VE, Hernadi L, Ito E, Dyakonova TL, Chistopolsky IA, Zakharov IS, et al. The activity of isolated neurons and the modulatory state of an isolated nervous system represent a recent behavioural state. J Exp Biol. 2015;218(8):1151–8. 10.1242/jeb.111930 25714568

[16] Rose RM, Benjamin PR. The relationship of the central motor pattern to the feeding cycle of Lymnaea stagnalis. J Exp Biol. 1979;80:137–63. 501275 10.1242/jeb.80.1.137

[17] Ferguson GP, Benjamin PR. The whole-body withdrawal response of Lymnaea stagnalis. I. Identification of central motoneurones and muscles. J Exp Biol. 1991;158:63–95. 1919418 10.1242/jeb.158.1.63

[18] Pirger Z, Crossley M, László Z, Naskar S, Kemenes G, O’Shea M, et al. Interneuronal mechanism for Tinbergen’s hierarchical model of behavioral choice. Curr Biol. 2014 9;24(17):2018–24. 10.1016/j.cub.2014.07.044 25155505 PMC4159561

[19] Staras K, Kemenes G, Benjamin PR. Pattern-generating role for motoneurons in a rhythmically active neuronal network. J Neurosci. 1998;18(10):3669–88. 10.1523/JNEUROSCI.18-10-03669.1998 9570798 PMC6793163

[20] Fujisaki Y, Matsuo R. Context-Dependent Passive Avoidance Learning in the Terrestrial Slug Limax. Zoolog Sci. 2017 12;34(6):532–7. 10.2108/zs170071 29219042

[21] Kimura T, Toda S, Sekiguchi T, Kirino Y. Behavioral modulation induced by food odor aversive conditioning and its influence on the olfactory responses of an oscillatory brain network in the slug Limax marginatus. Learn Mem. 1998;4(5):365–75. 10.1101/lm.4.5.365 10701876

[22] Kasai Y, Watanabe S, Kirino Y, Matsuo R. The procerebrum is necessary for odor-aversion learning in the terrestrial slug Limax valentianus. Learn Mem. 2006;13(4):482–8. 10.1101/lm.257606 16847307 PMC1538926

[23] Kleinfeld D, Delaney KR, Fee MS, Flores JA, Tank DW, Gelperin A. Dynamics of propagating waves in the olfactory network of a terrestrial mollusk: an electrical and optical study. J Neurophysiol. 1994 9;72(3):1402–19. 10.1152/jn.1994.72.3.1402 7807221

[24] Ermentrout B, Wang JW, Flores J, Gelperin A. Model for transition from waves to synchrony in the olfactory lobe of Limax. J Comput Neurosci. 2004;17(3):365–83. 10.1023/B:JCNS.0000044877.21949.44 15483397

[25] Panchina Y, Kelmanson I, Matz M, Lukyanov K, Usman N, Lukyanov S. A ubiquitous family of putative gap junction molecules [2]. Curr Biol. 2000;10(13):473–4. 10.1016/s0960-9822(00)00576-5 10898987

[26] Kelmanson IV., Shagin DA, Usman N, Matz M V., Lukyanov SA, Panchin Y V. Altering electrical connections in the nervous system of the pteropod mollusc Clione limacina by neuronal injections of gap junction mRNA. Eur J Neurosci. 2002;16(12):2475–6. 10.1046/j.1460-9568.2002.02423.x 12492443

[27] Mersman BA, Jolly SN, Lin Z, Xu F. Gap Junction Coding Innexin in Lymnaea stagnalis: Sequence Analysis and Characterization in Tissues and the Central Nervous System. Front Synaptic Neurosci. 2020;12:1. 10.3389/fnsyn.2020.00001 32158385 PMC7052179

[28] Moroz LL, Edwards JR, Puthanveettil SV., Kohn AB, Ha T, Heyland A, et al. Neuronal Transcriptome of Aplysia: Neuronal Compartments and Circuitry. Cell. 2006;127(7):1453–67. 10.1016/j.cell.2006.09.052 17190607 PMC4024467

[29] Sadamoto H, Takahashi H, Okada T, Kenmoku H, Toyota M, Asakawa Y. De novo sequencing and transcriptome analysis of the central nervous system of Mollusc Lymnaea stagnalis by deep RNA sequencing. PLoS One. 2012;7(8). 10.1371/journal.pone.0042546 22870333 PMC3411651

[30] Albertin CB, Simakov O, Mitros T, Wang ZY, Pungor JR, Edsinger-Gonzales E, et al. The octopus genome and the evolution of cephalopod neural and morphological novelties. Nature. 2015;524(7564):220–4. 10.1038/nature14668 26268193 PMC4795812

[31] Liu C, Zhang Y, Ren Y, Wang H, Li S, Jiang F, et al. The genome of the golden apple snail Pomacea canaliculata provides insight into stress tolerance and invasive adaptation. Gigascience. 2018 9;7(9). 10.1093/gigascience/giy101 30107526 PMC6129957

[32] Cai H, Li Q, Fang X, Li J, Curtis NE, Altenburger A, et al. Data descriptor: A draft genome assembly of the solar-powered sea slug elysia chlorotica. Sci Data. 2019;6:1–13. 10.1038/s41597-018-0005-2 30778257 PMC6380222

[33] Kumar S, Stecher G, Li M, Knyaz C, Tamura K. MEGA X: Molecular evolutionary genetics analysis across computing platforms. Mol Biol Evol. 2018;35(6):1547–9. 10.1093/molbev/msy096 29722887 PMC5967553

[34] Blom N, Sicheritz-Pontén T, Gupta R, Gammeltoft S, Brunak S. Prediction of post-translational glycosylation and phosphorylation of proteins from the amino acid sequence. Proteomics. 2004;4(6):1633–49. 10.1002/pmic.200300771 15174133

[35] Xue Y, Liu Z, Gao X, Jin C, Wen L, Yao X, et al. GPS-SNO: Computational prediction of protein s-nitrosylation sites with a modified GPS algorithm. PLoS One. 2010;5(6):1–7. 10.1371/journal.pone.0011290 20585580 PMC2892008

[36] Lowenthal MS, Davis KS, Formolo T, Kilpatrick LE, Phinney KW. Identification of Novel N-Glycosylation Sites at Noncanonical Protein Consensus Motifs. J Proteome Res. 2016;15(7):2087–101. 10.1021/acs.jproteome.5b00733 27246700 PMC5100817

[37] Kaji H, Kamiie JI, Kawakami H, Kido K, Yamauchi Y, Shinkawa T, et al. Proteomics reveals n-linked glycoprotein diversity in Caenorhabditis elegans and suggests an atypical translocation mechanism for integral membrane proteins. Mol Cell Proteomics. 2007;6(12):2100–9. 10.1074/mcp.M600392-MCP200 17761667

[38] Viklund H, Elofsson A. OCTOPUS: improving topology prediction by two-track ANN-based preference scores and an extended topological grammar. Bioinformatics [Internet]. 2008 5 12;24(15):1662–8. Available from: 10.1093/bioinformatics/btn221 18474507

[39] Phelan P. Innexins: members of an evolutionarily conserved family of gap-junction proteins. Biochim Biophys Acta—Biomembr [Internet]. 2005 6 10 [cited 2020 Jan 24];1711(2):225–45. Available from: 10.1016/j.bbamem.2004.10.004 15921654

[40] Suchyna TM, Xu LX, Gao F, Fourtner CR, Nicholson BJ. Identification of a proline residue as a transduction element involved in voltage gating of gap junctions. Nature. 1993 10;365(6449):847–9. 10.1038/365847a0 8413670

[41] Ri Y, Ballesteros JA, Abrams CK, Oh S, Verselis VK, Weinstein H, et al. The role of a conserved proline residue in mediating conformational changes associated with voltage gating of Cx32 gap junctions. Biophys J [Internet]. 1999;76(6):2887–98. Available from: 10.1016/S0006-3495(99)77444-8 10354417 PMC1300261

[42] Oshima A. Structure of an innexin gap junction channel and cryo-EM sample preparation. Reprod Syst Sex Disord. 2017 12;66(6):371–9. 10.1093/jmicro/dfx035 29036409 PMC6084585

[43] Panchin YV. Evolution of gap junction proteins—The pannexin alternative. J Exp Biol. 2005;208(8):1415–9. 10.1242/jeb.01547 15802665

[44] Gong XQ, Nakagawa S, Tsukihara T, Bai D. A mechanism of gap junction docking revealed by functional rescue of a human-disease-linked connexin mutant. J Cell Sci. 2013;126(14):3113–20. 10.1242/jcs.123430 23687377

[45] Bai D, Yue B, Aoyama H. Crucial motifs and residues in the extracellular loops influence the formation and specificity of connexin docking. Biochim Biophys Acta—Biomembr. 2018;1860(1):9–21. 10.1016/j.bbamem.2017.07.003 28693896

[46] Turnbull MW, Hasegawa DK, Turnbull MW. Recent Findings in Evolution and Function of Insect Innexins. Author ‘ s personal copy Recent findings in evolution and function of insect innexins. 2014;(March). 10.1016/j.febslet.2014.03.006 24631533

[47] Baranova A, Ivanov D, Petrash N, Pestova A, Skoblov M, Kelmanson I, et al. The mammalian pannexin family is homologous to the invertebrate innexin gap junction proteins. Genomics [Internet]. 2004 4 1 [cited 2020 Jan 24];83(4):706–16. Available from: 10.1016/j.ygeno.2003.09.025 15028292

[48] Eastman SD, Chen THP, Falk MM, Mendelson TC, Iovine MK. Phylogenetic analysis of three complete gap junction gene families reveals lineage-specific duplications and highly supported gene classes. Genomics. 2006;87(2):265–74. 10.1016/j.ygeno.2005.10.005 16337772

[49] Sanchez-pupo RE. N-Glycosylation Regulates Pannexin 2 Localization but Is Not Required for Interacting with Pannexin 1. 2018;1–18. 10.3390/ijms19071837 PMC6073767 29932112

[50] Penuela S, Bhalla R, Nag K, Laird DW. Glycosylation Regulates Pannexin Intermixing and Cellular Localization. Mol Biol Cell. 2009;20:4313–23. 10.1091/mbc.e09-01-0067 19692571 PMC2762227

[51] Boassa D, Qiu F, Dahl G, Sosinsky G. Trafficking dynamics of glycosylated pannexin1 proteins. Cell Commun Adhes. 2008;15(1–2):119–32. 18649184 10.1080/15419060802013885 PMC2528835

[52] Lohman AW, Weaver JL, Billaud M, Sandilos JK, Griffiths R, Straub AC, et al. S-nitrosylation inhibits pannexin 1 channel function. J Biol Chem. 2012;287(47):39602–12. 10.1074/jbc.M112.397976 23033481 PMC3501028

[53] Straub AC, Billaud M, Johnstone SR, Best AK, Yemen S, Dwyer ST, et al. Compartmentalized connexin 43 s-nitrosylation/denitrosylation regulates heterocellular communication in the vessel wall. Arterioscler Thromb Vasc Biol. 2011 2;31(2):399–407. 10.1161/ATVBAHA.110.215939 21071693 PMC3056333

[54] Retamal MA, Yin SY, Altenberg GA, Reuss L. Modulation of Cx46 hemichannels by nitric oxide. Am J Physiol—Cell Physiol. 2009;296(6). 10.1152/ajpcell.00054.2009 19357237 PMC2692414

[55] Sakura M, Kabetani M, Watanabe S, Kirino Y. Impairment of olfactory discrimination by blockade of nitric oxide activity in the terrestrial slug Limax valentianus. Neurosci Lett. 2004;370(2–3):257–61. 10.1016/j.neulet.2004.08.025 15488334

[56] Gelperin A, Kleinfeld D, Denk W, Cooke IR. Oscillations and gaseous oxides in invertebrate olfaction. J Neurobiol. 1996 5;30(1):110–22. 10.1002/(SICI)1097-4695(199605)30:1&lt;110::AID-NEU10&gt;3.0.CO;2-Q 8727987

[57] Yabumoto T, Takanashi F, Kirino Y, Watanabe S. Nitric oxide is involved in appetitive but not aversive olfactory learning in the land mollusk Limax valentianus. Learn Mem. 2008;15(4):229–32. 10.1101/lm.936508 18385478

[58] Kobayashi S, Sadamoto H, Ogawa H, Kitamura Y, Oka K, Tanishita K, et al. Nitric oxide generation around buccal ganglia accompanying feeding behavior in the pond snail, Lymnaea stagnalis. Neurosci Res. 2000;38(1). 10.1016/s0168-0102(00)00136-x 10997575

[59] Sadamoto H, Hatakeyama D, Kojima S, Fujito Y, Ito E. Histochemical study on the relation between NO-generative neurons and central circuitry for feeding in the pond snail, Lymnaea stagnalis. Neurosci Res. 1998;32(1). 10.1016/s0168-0102(98)00066-2 9831252

[60] Fujie S, Aonuma H, Ito I, Gelperin A, Ito E. The Nitric Oxide/Cyclic GMP Pathway in the Olfactory Processing System of the Terrestrial Slug Limax marginatus. Zoolog Sci [Internet]. 2002 1 1;19(1):15–26. Available from: 10.2108/zsj.19.15 12025400

[61] Laird DW. Connexin phosphorylation as a regulatory event linked to gap junction internalization and degradation. Biochim Biophys Acta—Biomembr. 2005;1711(2 SPEC. ISS.):172–82. 10.1016/j.bbamem.2004.09.009 15955302

[62] Kanemitsu MY, Jiang W, Eckhart W. Cdc2-mediated phosphorylation of the gap junction protein, connexin43, during mitosis. Cell Growth Differ. 1998;9(1):13–21. 9438384

[63] Ragan C, Goodall GJ, Shirokikh NE, Preiss T. Insights into the biogenesis and potential functions of exonic circular RNA. Sci Rep [Internet]. 2019;9(1):1–18. Available from: 10.1038/s41598-018-37037-0 30765711 PMC6376117

[64] Westholm JO, Miura P, Olson S, Shenker S, Joseph B, Sanfilippo P, et al. Genome-wide Analysis of Drosophila Circular RNAs Reveals Their Structural and Sequence Properties and Age-Dependent Neural Accumulation. Cell Rep [Internet]. 2014;9(5):1966–80. Available from: 10.1016/j.celrep.2014.10.062 25544350 PMC4279448

[65] Memczak S, Jens M, Elefsinioti A, Torti F, Krueger J, Rybak A, et al. Circular RNAs are a large class of animal RNAs with regulatory potency. Nature. 2013 3;495(7441):333–8. 10.1038/nature11928 23446348

[66] You X, Vlatkovic I, Babic A, Will T, Epstein I, Tushev G, et al. Neural circular RNAs are derived from synaptic genes and regulated by development and plasticity. Nat Neurosci. 2015;18(4):603–10. 10.1038/nn.3975 25714049 PMC4376664

[67] Dudekula DB, Panda AC, Grammatikakis I, De S, Abdelmohsen K, Gorospe M. Circinteractome: A web tool for exploring circular RNAs and their interacting proteins and microRNAs. RNA Biol [Internet]. 2016;13(1):34–42. Available from: 10.1080/15476286.2015.1128065 26669964 PMC4829301

[68] Calkins TL, Woods-Acevedo MA, Hildebrandt O, Piermarini PM. The molecular and immunochemical expression of innexins in the yellow fever mosquito, Aedes aegypti: Insights into putative life stage- and tissue-specific functions of gap junctions. Comp Biochem Physiol Part—B Biochem Mol Biol. 2015 5;183:11–21. 10.1016/j.cbpb.2014.11.013 25585357 PMC4380524

[69] Naser Al Deen N, AbouHaidar M, Talhouk R. Connexin43 as a Tumor Suppressor: Proposed Connexin43 mRNA-circularRNAs-microRNAs Axis Towards Prevention and Early Detection in Breast Cancer. Front Med. 2019;6(August):1–8. 10.3389/fmed.2019.00192 31555649 PMC6724403

[70] Hansen TB, Jensen TI, Clausen BH, Bramsen JB, Finsen B, Damgaard CK, et al. Natural RNA circles function as efficient microRNA sponges. Nature [Internet]. 2013;495(7441):384–8. Available from: 10.1038/nature11993 23446346

[71] Yang Y, Fan X, Mao M, Song X, Wu P, Zhang Y, et al. Extensive translation of circular RNAs driven by N 6 -methyladenosine. Cell Res. 2017;27(5):626–41. 10.1038/cr.2017.31 28281539 PMC5520850

[72] Wang Y, Wang Z. Efficient backsplicing produces translatable circular mRNAs. Rna. 2015;21(2):172–9. 10.1261/rna.048272.114 25449546 PMC4338345

[73] Smyth JW, Shaw RM. Autoregulation of connexin43 gap junction formation by internally translated isoforms. Cell Rep [Internet]. 2013;5(3):611–8. Available from: 10.1016/j.celrep.2013.10.009 24210816 PMC3898934

[74] Esseltine JL, Laird DW. Next-Generation Connexin and Pannexin Cell Biology. Trends Cell Biol [Internet]. 2016;26(12):944–55. Available from: 10.1016/j.tcb.2016.06.003 27339936

[75] Kotini M, Barriga EH, Leslie J, Gentzel M, Rauschenberger V, Schambon A, et al. Gap junction protein Connexin-43 is a direct transcriptional regulator of N-cadherin in vivo. Nat Commun [Internet]. 2018;9(1). Available from: 10.1038/s41467-018-06368-x PMC6155008 30242148

